# Impact of pre-existing conditions on the severity of post-COVID syndrome among workers in healthcare and social services in Germany

**DOI:** 10.1186/s12995-024-00431-8

**Published:** 2024-08-01

**Authors:** Tiana Barnekow, Claudia Peters, Madeleine Dulon, Albert Nienhaus

**Affiliations:** 1 Competence Centre for Epidemiology and Health Services Research for Healthcare Professionals (CVcare), University Medical Centre Hamburg-Eppendorf (UKE), 20246 Hamburg, Germany; 2 Department of Occupational Medicine, Hazardous Substances and Public Health, Institution for Statutory Accident Insurance and Prevention in the Healthcare and Welfare Services, 22089 Hamburg, Germany

**Keywords:** COVID-19, post-COVID, Long COVID, Healthcare workers, Social workers, Pre-existing conditions

## Abstract

**Background:**

The underlying mechanisms of post-COVID syndrome (PCS) are still not fully understood. While pre-existing conditions have been described as a risk factor for severe PCS in the general population, data specific to different occupational groups in this context is lacking. This study aimed to investigate the impact of pre-existing conditions on severe post-COVID syndrome among the occupational group of healthcare and social services employees.

**Methods:**

Baseline data from a longitudinal, observational study were analysed. In February 2021, health workers who had a COVID-19 infection in 2020 were surveyed about sequelae of the infection. Factors influencing severe PCS with at least one persistent symptom categorised as severe were subjected to a multivariate logistic regression analysis.

**Results:**

Of a total of 2,053 participants, 21.5% had severe PCS. Underlying respiratory (OR 1.94; CI 1.44–2.61), cardiovascular (OR 1.35; CI 1.04–1.77) and urogenital (OR 1.79; CI 1.10–2.91) disease were risk factors for severe PCS overall. Respiratory and mental illnesses had a statistically significant impact on persistent fatigue/exhaustion, concentration/memory difficulties and shortness of breath categorised as severe. Urogenital disease was associated with severe fatigue/exhaustion. Other significant risk factors for severe PCS were female sex, smoking, physical exercise and hospitalisation due to COVID-19 infection.

**Conclusion:**

Workers in healthcare and social services with pre-existing conditions may face a higher risk of developing severe PCS. Additional analyses performed as part of the longitudinal study will show if and how this result changes over time.

**Supplementary Information:**

The online version contains supplementary material available at 10.1186/s12995-024-00431-8.

## Introduction

The COVID-19 pandemic has placed significant strain on the global healthcare system. Being at the frontline of patient care, health workers faced high risks of contracting COVID-19, particularly within the first six months of the pandemic [1, 2].

While much attention has been given to the acute phase of COVID-19 infections, there is ongoing concern regarding the long-term health consequences of COVID-19. Post-COVID syndrome is defined as the continuation of symptoms twelve weeks after a suspected or confirmed COVID-19 infection or the development of new symptoms during this period [3]. The syndrome is associated with a wide range of physical, neurocognitive and psychological symptoms and can have a significant negative impact on the personal and professional lives of patients. Compared to the general population, post-COVID patients have a significantly lower health-related quality of life [4, 5] and, according to a meta-analysis, 14.1% of those affected remain unable to work [6]. Data from health insurers in Germany confirm that insured individuals with a post-COVID syndrome diagnosis have an above average number of days being absent from work [7].

Data on post-COVID prevalence are heterogeneous. In the general population, it is estimated that up to 45% of COVID-19 survivors experience persistent symptoms [8], while other authors estimate the prevalence of post-COVID syndrome at 6.5–12.7% [9, 10]. Reports on post-COVID prevalence among health workers in Europe range from 10% [11] to 16.3% [12].

Despite intense research efforts, the pathogenesis of the disease remains insufficiently understood. According to a meta-analysis, risk factors identified to date include hospitalisation due to COVID-19 infection, female sex, advanced age, high BMI and smoking [13]. Reinfections increase the risk [14], being fully vaccinated against SARS-CoV-2 reduces it [13]. Comorbidities have also been described as risk factors: Associations between underlying disease and post-COVID exist for asthma and chronic obstructive pulmonary disease (COPD) [13, 15–17], depression and anxiety disorders [13, 18, 19] and/or pre-existing psychological conditions in general [17], gastrointestinal disease [18], diabetes [13, 15], autoimmune disorders/immunosuppression [13, 18, 20], hypertension [21] and ischaemic heart disease [13]. Data on risk factors for severe post-COVID syndrome in particular are still scarce. Current known risk factors for severe post-COVID include female sex, coagulation disorder and coronary heart disease [4], pre-existing neurological and cardiovascular disease [22] as well as pre-existing respiratory disease [5].

Health workers with post-COVID syndrome pose a unique patient group: Due to the challenge of finding a balance between being a patient and health professional at the same time, these employees report struggles in receiving appropriate medical attention for their post-COVID complaints [23]. Despite these challenges, there is limited literature available on post-COVID among health workers, and data on risk factors specifically for severe post-COVID within this group is lacking. This study aimed to investigate the impact of pre-existing illnesses on severe post-COVID syndrome among workers in healthcare and social services.

## Methods

### Study design and study population

In a cross-sectional study design, health workers were surveyed about how they had been affected by their COVID-19 infection. These evaluations are part of the baseline questionnaire in a longitudinal study of insured persons carried out by the German Institution for Statutory Accident Insurance in the Health and Welfare Services (BGW). The study population included all individuals with BGW policies in the districts of Cologne and Dresden who reported a case of COVID-19 infection suspected to be resulting from occupational exposure before 31.12.2020. BGW policies apply to employees in both healthcare and social services. As the majority of reported COVID-19 infection cases involved healthcare employees, both occupational groups will be collectively referred to as ‘health workers’ hereafter. A total of 4,325 insured persons were contacted by mail in February 2021 and invited to take part in the study. The included information contained in-depth descriptions about the study objectives, the study schedule and data privacy. The inclusion criterion for the study was a COVID-19 infection that was suspected to have originated in the workplace and that was confirmed by PCR test and/or the presence of symptoms. If the participant had not been infected with COVID-19 infection, or if they had a limited ability to read and write or understand German, they were excluded from the study. All participants gave their written consent to participate in the study. A detailed description of the study procedure and population can be found in a previous paper by Peters et al. [24]. This study was approved by the Ethics Committee of the Medical Association of Hamburg (2021-10463-BO-ff).

### Data collection

A paper and pencil survey was utilized to collect sociodemographic data, information about lifestyle factors such as height, weight, smoking and physical activity levels, in addition to information about each participant’s occupational situation. Other questions included details on the participant’s medical history: Pre-existing conditions were divided into categories and a distinction was made between “self-diagnosis”, “medical diagnosis” and “not applicable” for each condition. Regarding the initial COVID-19 infection, details were obtained on the date and type of test used, as well as on acute and persisting symptoms. Information about more than ten symptoms was collected for both the acute infection and for the time of the survey. Participants could also add information about other symptoms in their own words. Where applicable, symptoms could be assigned a degree of severity (mild, moderate or severe). Other data was collected regarding the treatment of the COVID-19 infection (inpatient or outpatient, intensive care ward (ICU)) and rehabilitation measures related to the infection, as well as the participant’s ability to work and their physical and mental health.

### Definition of the studied pathology

This cross-sectional study investigated the impact of underlying disease on severe chronic sequelae of COVID-19 infections. Participants were considered to have severe post-COVID syndrome if their symptoms had persisted for longer than 12 weeks and at least one of the symptoms on the survey had been categorised as “severe”. The term “post-COVID syndrome” or the abbreviation PCS is used below in line with the definitions published by the WHO [3] and Association of the Scientific Medical Societies in Germany (AWMF) [25].

### Statistical methods

The statistical analysis was carried out using IBM SPSS (version 27.0.0.0). The data underwent descriptive analysis and was presented with absolute and relative frequencies. Significant differences between the groups were calculated using the chi-squared test, or, where applicable, with Fisher’s exact test. Only underlying disease that had been clinically diagnosed was taken into account, otherwise it was discounted. Fields that were left blank in the categories of smoking, physical exercise, underlying disease and symptoms were considered equivalent to a not applicable response. Multivariate logistic regression analyses were carried out in order to identify risk factors. Severe post-COVID syndrome and the five most common severe PCS symptoms were defined as dependent variables. Predictor variables included cardiovascular disease, respiratory disease, mental disorders, urogenital disease and hormonal/metabolic disease. Confounding variables included sex, age, BMI category, smoking and hospitalisation (inpatient, ICU). The Chance criterion design was used for modelling [26]: Variables were removed from the model if their p-value was greater than 0.1 and the removal of the variable did not affect significant results (*p* < 0.1) of other variables. Each model was adjusted for age and sex, regardless of their p-value. A p-value of ≤ 0.05 was deemed statistically significant.

## Results

### Study population

Out of the 4,325 eligible individuals contacted, 2,053 participants were included in the study (response rate of approximately 50%). A total of 554 individuals (12.8%) did not meet the inclusion criteria and were subsequently excluded from the study. In-depth details on the selection process for this study population have been previously described by Peters et al. [24].

The majority of participants were female (81.7%) and aged between 50 and 59 years (36.3%). Of the participants, 15.9% were smokers, 32.4% were overweight using WHO criteria [27] and 23.6% were obese. The majority of participants regularly engaged in physical activity for at least one hour a week (68%). With regard to the COVID-19 infection, 4.8% of participants had been treated as inpatients. 1.8% had been treated on an ICU. 55.5% of participants had a clinically diagnosed pre-existing condition. The most common pre-existing condition was cardiovascular disease (25.6%), followed by hormonal/metabolic disease (23.7%) and mental disorders (12.3%) (Table 1).

**Table 1:** Characteristics of the study population (*n* = 2053)

		*n*	%
Sex	Male	377	18.3
	Female	1,676	81.7
Age in years	< 30	217	10.6
	30–39	326	15.9
	40–49	384	18.7
	50–59	745	36.3
	60+	381	18.6
BMI	Underweight	31	1.5
	Normal weight	872	42.5
	Overweight	666	32.4
	Obesity	484	23.6
Smoking		327	15.9
Physical exercise	No physical exercise	655	31.9
	1 h/week	486	23.6
	2–3 h/week	587	28.6
	> 3 h/week	325	15.8
Hospitalisation	Inpatient	99	4.8
	ICU	36	1.8
Clinically diagnosed conditions	At least one condition	1,138	55.5
	Cardiovascular diseases	525	25.6
	Respiratory diseases	251	12.2
	Mental disorders	252	12.3
	Urogenital diseases	84	4.1
	Hormonal/metabolic diseases	486	23.7

BMI: Body mass index, ICU: Intensive care unit

### Prevalence of post-COVID syndrome and group differences

At the time of data collection, 68.4% of participants had post-COVID syndrome with persistent symptoms > 12 weeks after a COVID-19 infection. 33.9% of those with PCS (21.5% of the total study population) were classed as having severe post-COVID with at least one severe symptom (Table S1). Compared to the control group, respondents with severe PCS were more likely to be female (87.9% vs. 79.8%) and older (61.3% vs. 52.9% ≥50 years). Obesity and smoking were also more common in the group with severe PCS. Participants affected by PCS were more likely to have been treated as an inpatient or on an ICU for a COVID-19 infection than those in the control group. Respondents with severe PCS were much more likely to have underlying disease, both overall (66.6% vs. 52.1%) and in each diagnosis category in the questionnaire (Table 2).

**Table 2:** Characteristics of participants with severe post-COVID syndrome (PCS, at least one severe symptom > 12 weeks after COVID-19 infection) and participants without severe PCS.

		Severe post-COVID (*n* = 442, 21,5%)		No severe post-COVID (*n* = 1611, 78,5%)		*p*-value
		*n*	%	*n*	%	
Sex	Male	55	12.4	322	20.0	< 0.001
	Female	387	87.6	1,289	80.0	
Age in years	< 30	44	10.0	173	10.7	0.012
	30–39	52	11.8	274	17.0	
	40–49	76	17.2	308	19.1	
	50–59	188	42.5	557	34.6	
	60+	82	18.6	299	18.6	
BMI	Underweight	13	2.9	18	1.1	< 0.001
	Normal weight	142	32.1	730	45.3	
	Overweight	148	33.5	518	32.1	
	Obesity	139	31.4	345	21.4	
Smoking		85	19.2	242	15.0	0.040
Physical exercise	No physical exercise	149	33.7	506	31.4	0.742
	1 h/week	104	23.5	382	23.7	
	2–3 h/week	125	28.3	462	28.7	
	> 3 h/week	64	14.5	261	16.2	
Hospitalisation	Inpatient	36	8.1	63	3.9	< 0.001
	ICU	19	4.3	17	1.1	
Clinically diagnosed conditions	At least one condition	396	67.0	842	52.3	< 0.001
	Cardiovascular diseases	152	34.4	373	23.2	< 0.001
	Respiratory diseases	91	20.6	160	9.9	< 0.001
	Mental disorders	79	17.9	173	10.7	< 0.001
	Urogenital diseases	31	7.0	53	3.3	< 0.001
	Hormonal/metabolic diseases	130	29.4	356	22.1	0.002

BMI: Body mass index; ICU: Intensive care unit

### Prevalence of persistent symptoms with PCS

The five most common persistent symptoms, regardless of the degree of severity, were fatigue/exhaustion (82.8%), concentration/memory difficulties (70.7%), shortness of breath (56.4%), headache (41.3%) and loss of sense of smell/taste (28.6%). The five most common persistent symptoms classed as severe were fatigue/exhaustion (17.2%), concentration/memory difficulties (9.5%), loss of sense of smell/taste (9.0%), shortness of breath (7.3%) and pain in the limbs (5.5%) (Table S1).

**Fig. 1:**
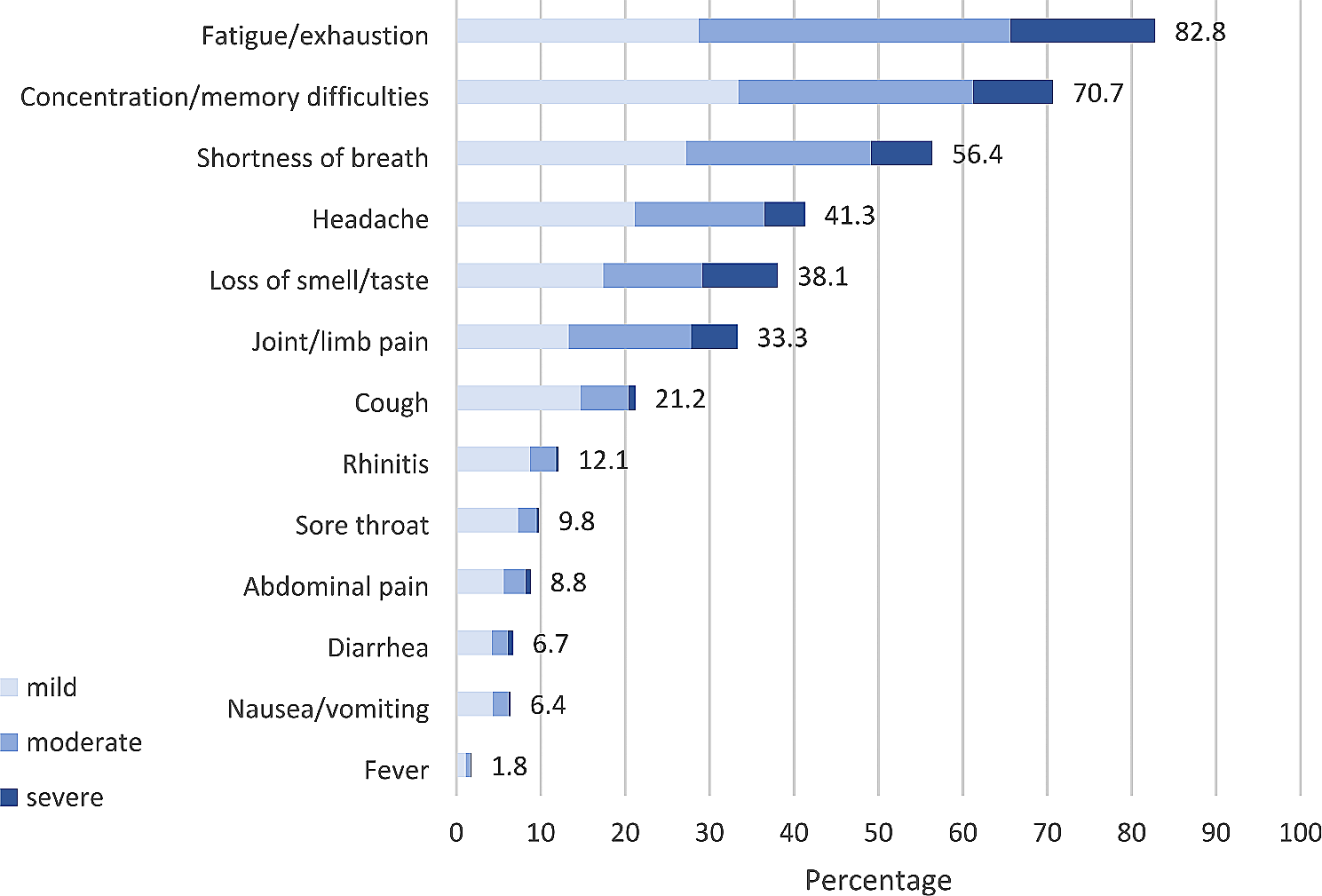
Persistent symptoms in participants with PCS (n = 1,404, 68.4%). Percentage distribution of degrees of severity: mild, moderate and severe along with the overall prevalence for each symptom.

Figure 1 provides an overview of persistent symptoms among those with PCS, differentiated by severity.

### Risk factors for severe post-COVID

Table 3 presents the results of the regression analyses for severe PCS overall, as well as for the most common persistent symptoms associated with the COVID-19 infection. Investigated predictive variables included age and sex, clinically diagnosed pre-existing conditions, BMI, smoking and hospitalisation as a result of the COVID-19 infection.

**Table 3:** Predictors for severe PCS overall, as well as for the three most frequent severe symptoms

	Severe post-COVID overall (at least one severe symptom) (*n* = 442, 21.5%)	Severe fatigue/exhaustion (*n* = 242, 11.7%)	Severe concentration/memory difficulties (*n* = 133, 4.6%)	Severe shortness of breath (*n* = 102, 5.0%)
	OR (95% CI; *p*-value)	OR (95% CI; *p*-value)	OR (95% CI; *p*-value)	OR (95% CI; *p*-value)
**Sex (ref: men)**	OR 1.78 (1.29–2.45; *p* < 0.001)	OR 1.64 (1.09–2.49; *p* = 0.019)	OR 1.73 (0.99–3.05; *p* = 0.056)	OR 2.10 (1.06–4.17; *p* = 0.034)
**Age in years**	OR 1.00 (0.99–1.01; *p* = 0.950)	OR 1.00 (0.99–1.02; *p* = 0.597)	OR 0.99 (0.97-1.00; *p* = 0.098)	OR 1.01 (0.99–1.02; *p* = 0.526)
**Cardiovascular diseases**	OR 1.35 (1.04–1.77; *p* = 0.025)	-	-	-
**Respiratory diseases**	OR 1.94 (1.44–2.61; *p* < 0.001)	OR 1.68 (1.17–2.42; *p* = 0.005)	OR 1.63 (1.03–2.58; *p* = 0.039)	OR 4.21 (2.71–6.54; *p* < 0.001)
**Mental disorders**	OR 1.36 (0.99–1.85; *p* = 0.053)	OR 1.93 (1.35–2.76; *p* < 0.001)	OR 2.53 (1.64–3.89; *p* < 0.001)	OR 1.67 (1.01–2.78; *p* = 0.047)
**Urogenital diseases**	OR 1.79 (1.10–2.91; *p* = 0.018)	OR 1.87 (1.07–3.28; *p* = 0.029)	-	-
**BMI** (ref.: normal weight)	-	-	-	-
Underweight	OR 3.30 (1.56–6.99; *p* = 0.002)	OR 3.95 (1.72–9.08; *p* = 0.001)	OR 2.55 (0.82–7.90; *p* = 0.104)	OR 4.95 (1.53–16.05; *p* = 0.008)
Overweight	OR 1.38 (1.06–1.81; *p* = 0.017)	OR 1.47 (1.04–2.07; *p* = 0.029)	OR 1.19 (0.75–1.90; *p* = 0.461)	OR 1.84 (1.04–3.26; *p* = 0.036)
Obesity	OR 1.56 (1.16–2.09; *p* = 0.003)	OR 1.72 (1.20–2.46; *p* = 0.003)	OR 1.79 (1.12–2.88; *p* = 0.016)	OR 2.76 (1.58–4.83; *p* < 0.001)
**Physical exercise** (ref: no exercise)	-	-	-	-
1 h/week	-	-	OR 1.32 (0.81–2.16; *p* = 0.266)	-
2–3 h/week	-	-	OR 1.76 (1.11–2.82; *p* = 0.017)	-
> 3 h/week	-	-	OR 0.95 (0.50–1.83; *p* = 0.888)	-
**Smoking**	OR 1.46 (1.10–1.95; *p* = 0.008)	OR 1.53 (1.07–2.18; *p* = 0.019)	OR 1.69 (1.09–2.63; *p* = 0.019)	-
**Hospitalisation** (ref: no hospitalisation)	-	-	-	-
Inpatient	OR 1.93 (1.24–3.02; *p* = 0.004)	OR 2.22 (1.32–3.71; *p* = 0.002)	OR 2.40 (1.28–4.50; *p* = 0.007)	OR 2.58 (1.35–4.93; *p* = 0.004)
ICU	OR 4.26 (2.10–8.63; *p* < 0.001)	OR 4.29 (2.03–9.05; *p* < 0.001)	OR 2.28 (0.81–6.43; *p* = 0.121)	OR 3.22 (1.18–8.80; *p* = 0.022)

OR: odds ratio (95% confidence intervall), ref: reference, ICU: intensive care unit

Each model was adjusted for sex and age regardless of the *p*-value. Empty fields: The variable was not significant and thus not included in the adjusted model

Other pre-existing conditions showed no statistically significant impact on any of the outcomes

#### Effect of underlying disease on severe post-COVID

Pre-existing conditions had varying effects on severe PCS and severe persistent symptoms. Respiratory disease, mental disorders, cardiovascular disease and urogenital conditions were identified as risk factors for severe PCS (Table 3).

Respiratory disease in particular was identified as increasing the risk of severe PCS: The risk of participants with underlying respiratory disease developing severe PCS was twice as high overall. Additionally, their risk of developing severe fatigue and severe concentration/memory problems was more than 1.5 times higher. The risk of severe shortness of breath was more than four times higher compared to participants without respiratory disease.

Mental disorders did not have an overall influence on severe PCS but did affect individual symptoms. Underlying mental disorders doubled the risk of severe concentration/memory problems. The risk of severe fatigue/exhaustion (OR 1.93) and severe shortness of breath (OR 1.67) was also statistically significantly higher for people with mental disorders.

Urogenital disorders were associated with nearly double the risk of severe PCS overall as well as severe fatigue/exhaustion.

Cardiovascular disease increased the overall risk of severe PCS (OR 1.35), although this result could not be confirmed for individual severe symptoms.

Other pre-existing conditions considered did not have any influence on the risk of developing severe PCS or severe PCS symptoms.

#### Other factors contributing to severe post-COVID

The risk of women suffering from severe PCS overall was almost twice as high compared to men. The risk of severe fatigue/exhaustion (OR 1.64) and severe shortness of breath (OR 2.10) was also significantly higher for women. None of the age categories had any influence on the risk of developing severe PCS or severe PCS symptoms (Table 3).

Body mass index was associated with a significantly higher risk for the outcomes in the study, particularly being underweight (OR 2.55–4.95) and obese, (OR 1.56–2.76), although being overweight (OR 1.38–1.84) increased the risk in comparison to normal weight as well. Smokers had a 1.5 times higher risk for severe PCS overall and severe fatigue/exhaustion, as well as a 1.7 times higher risk for severe concentration/memory difficulties. Being physically active for 2–3 h a week increased the risk of severe concentration/memory difficulties compared to no physical exercise (Table 3).

Hospitalisation as a result of the COVID-19 infection could also be identified as a significant risk factor. The risk of severe PCS overall doubled with inpatient treatment and quadrupled with treatment on an ICU. Hospitalisation was also a risk factor for all of the persistent symptoms in the study: Treatment on an ICU increased the risk more significantly (OR 2.28–4.29) than inpatient treatment (OR 2.22–2.58) (Table 3).

The risk of severe persistent joint/limb pain (no table) was not influenced by underlying disease, but was associated with female sex (OR 4.56; 95% CI 1.64–12.68; *p* = 0.004), age (OR 1.03; 95% CI 1.01–1.06; *p* = 0.007) and being overweight/obese (OR 2.96; 95% CI 1.52–5.76; *p* = 0.001 and OR 3.95; 95% CI 2.04–7.67; *p* < 0.001, respectively). Inpatient treatment (OR 2.37; 95% CI 1.12–5.03; *p* = 0.024) and ICU treatment (OR 3.37; 95% CI 1.21–9.36; *p* = 0.020) also influenced the risk of severe joint/limb pain. There was no association between severe persistent loss of sense of smell/taste and the predictors in the study.

## Discussion

In this study of 2,053 health workers who had a COVID-19 infection in 2020, we identified several pre-existing conditions and other factors as predictors for severe post-COVID syndrome. Respiratory and cardiovascular disease were associated with an increased overall risk of severe PCS. Respiratory, mental and urogenital conditions were identified as risk factors for persistent fatigue/exhaustion, concentration/memory difficulties and severe shortness of breath. Other significant risk factors for severe PCS were female sex, abnormal BMI, smoking and hospitalisation due to COVID-19 infection.

### PCS symptoms

For individuals with PCS in our study, the most common persistent symptoms regardless of severity were fatigue/exhaustion, concentration/memory difficulties, shortness of breath, headache and loss of sense of smell/taste. This corresponds with the results from other studies on PCS in health workers. Multiple studies have observed fatigue/exhaustion and concentration difficulties as frequent symptoms [12, 28, 29]. Shortness of breath [12] and headache [29] have also already been described as common symptoms.

There is a lack of comparative studies available investigating PCS symptoms classified solely as severe. In our study, the most common persistent PCS symptoms categorised as severe were fatigue/exhaustion, loss of sense of smell/taste, concentration/memory difficulties, shortness of breath and pain in the limbs.

### Risk factors for severe post-COVID symptoms

To date, there have only been few studies investigating risk factors for PCS for different degrees of symptom severity. The results of our study build on this knowledge and provide additional findings regarding risk factors, both for severe post-COVID generally and for individual persistent symptoms classed as severe.

#### Pre-existing conditions as risk factors

Our study identified cardiovascular, respiratory, mental and urogenital conditions as risk factors for severe PCS. All four types of disease were found to be significantly associated with severe PCS symptoms in previous studies [4, 5, 22], thus confirming our findings. Our results also build upon the findings of other studies on pre-existing conditions as risk factors for PCS, which did not account for degree of severity: In terms of respiratory and mental disease, studies have already identified asthma [13, 18] and COPD [13], as well as depression/anxiety disorders [13, 18] and psychological disorders in general [16, 30] as predictive factors for PCS. For cardiovascular disease, previously described associations with an increased risk for PCS include hypertension [21] and ischaemic heart disease [13]. In terms of urogenital diseases, three studies investigated nephrological conditions but found no association with the risk of PCS [13, 18, 22]. It may be the case that the influence of other urogenital conditions was not accounted for in these studies, thus possibly explaining the contradiction with our results.

Our findings also extend the current knowledge on links between pre-existing conditions and individual symptoms in PCS: In our study, respiratory and mental illnesses were shown to be associated with a higher risk of severe fatigue/exhaustion, concentration/memory difficulties and shortness of breath. Two studies have already described similar links between fatigue in general and respiratory and/or mental disorders [31, 32]. Another study found an association between depression and neuropsychiatric symptoms [33].

Two studies described an association between PCS and metabolic disease in the case of diabetes [13, 15]. We couldn’t confirm this finding for severe hormonal/metabolic diseases in our study.

#### Other contributing factors

Female sex and hospitalisation are known risk factors for PCS, both for severe PCS specifically [4, 22] as well as PCS in general. For PCS in general, the association with female sex has been described both for the general population [13] and for healthcare workers in particular [12]. Furthermore, two studies identified hospitalisation as a risk factor for fatigue and cognitive impairment [34] and shortness of breath [35]. Another study identified links between female sex and fatigue in people with PCS [33]. We were able to observe all of the mentioned correlations for the respective severe symptoms in our study as well.

Smoking was previously identified as a risk factor for PCS in general [13], as well as for cognitive impairment [36–38] and fatigue [37] in patients with PCS. Similar correlations were found in our study for these severe symptoms. The link between smoking and shortness of breath, however, remains unclear. Menges et al. identified smoking as a risk factor for shortness of breath [35], whereas Trofor et al. found no such association [38]. In our study, smoking did not have any influence on the risk of developing severe shortness of breath.

Deviations from the normal weight range are also known as being a risk factor for PCS. This applies in particular to the overweight and obese categories: Such correlations have been described both for the general population [13] and for healthcare workers in particular [12]. Our study builds upon this knowledge for the overweight and obese categories regarding the risk for severe PCS, while also identifying being underweight as significant risk factor. Being underweight showed a stronger effect on risk than being overweight or obese. However, the significance of these results is potentially limited due to the small sample size of underweight participants and broad confidence intervals.

The current data on the influence of advancing age on PCS risk is inconsistent. In the meta-analysis by Tsampasian et al., age is shown to be a significant risk factor, although the authors emphasise the high level of heterogeneity among the studies reviewed [13]. The study by Štěpánek et al. on PCS in healthcare workers only showed a low correlation between age and risk of PCS [39]. Our study found no correlation between age and the risk of severe PCS.

### Strengths and limitations

To our knowledge, this is the first study to investigate pre-existing conditions as a risk factor for severe post-COVID syndrome in health workers. The study’s strengths include the large study population with participants from various areas of work from two locations in Germany. The high response rate was another positive factor.

There are some limitations of note. People with severe post-COVID symptoms may have been more motivated to take part in the study, which may have led to this group being overrepresented. The use of questionnaires in the study design poses a risk for several types of bias: The responses are subjective assessments that cannot be validated, which introduces potential response bias. Additionally, recall bias cannot be ruled out. Because only severe symptoms were assessed, some of the sub-groups in the study only have small sample sizes. This may affect the significance of the results. It is also not possible to determine whether the reported symptoms are directly linked to the COVID-19 infection or whether they occurred independently. The lack of studies investigating underlying disease as a risk factor for different degrees of severity of PCS makes it more difficult to compare the results.

### Conclusion

In this study, we were able to show that cardiovascular, mental, urogenital and, in particular, respiratory conditions may increase the risk of severe post-COVID among health workers. Accounting for underlying disease in therapy and rehabilitation measures for treating post-COVID may increase therapeutic success.

The findings of this study expand our knowledge of post-COVID among health workers and may help to improve prevention and rehabilitation measures. This study population continues to be monitored as part of the ongoing longitudinal study on the sequelae of COVID-19 infections among workers in healthcare and social services. Analysing future data from this longitudinal study may provide further information regarding the impact of underlying disease on severe post-COVID.

### Electronic supplementary material

Below is the link to the electronic supplementary material.

Supplementary Material 1

## Data Availability

Data are made available upon reasonable request to the corresponding author.
