# Supplementary material for: Impact of pre-existing conditions on the severity of post-COVID syndrome among workers in healthcare and social services in Germany
Source: J Occup Med Toxicol. 2024 Aug 1;19:32. doi: 10.1186/s12995-024-00431-8 (PMC11293209; doi:10.1186/s12995-024-00431-8)
Supplement: Supplementary file 1 — Supplementary Material 1 [file 12995_2024_431_MOESM1_ESM.docx]

**Impact of Pre-Existing Conditions on the Severity of Post-COVID Syndrome among Workers in Healthcare and Social Services in Germany**

Authors: Tiana Barnekow, Claudia Peters, Madeleine Dulon, Albert Nienhaus

Affiliation and e-mail address of corresponding author: Tiana Barnekow, t.barnekow@uke.de

**Supplementary material**

Supplementary table S1: Prevalence of PCS and severe PCS (n = 2,053) as well as prevalence and severity of symptoms in participants with PCS at the time of the survey (n = 1,404).

|  |  | **Total study population** | |
| --- | --- | --- | --- |
|  |  | n = 2,053 | % |
| Symptomatic | No | 530 | 25,8 |
|  | Yes | 1,523 | 74,2 |
| Post-COVID syndrome | No | 649 | 31,6 |
|  | Yes | 1,404 | 68,4 |
| Severe post-COVID syndrome  (at least one severe symptom) | No | 1,611 | 78,5 |
|  | Yes | 442 | 21,5 |
| **Symptoms** |  | **Post-COVID syndrome** | |
|  |  | n = 1404 | % |
| Fatigue/exhaustion | None | 241 | 17,2 |
|  | Mild | 403 | 28,7 |
|  | Moderate | 518 | 36,9 |
|  | Severe | 242 | 17,2 |
| Concentration/memory difficulties | None | 412 | 29,3 |
|  | Mild | 469 | 33,4 |
|  | Moderate | 390 | 27,8 |
|  | Severe | 133 | 9,5 |
| Shortness of breath | None | 612 | 43,6 |
|  | Mild | 382 | 27,2 |
|  | Moderate | 308 | 21,9 |
|  | Severe | 102 | 7,3 |
| Headache | None | 825 | 58,8 |
|  | Mild | 296 | 21,1 |
|  | Moderate | 216 | 15,4 |
|  | Severe | 67 | 4,8 |
| Loss of smell/taste | None | 870 | 62,0 |
|  | Mild | 244 | 17,4 |
|  | Moderate | 164 | 11,7 |
|  | Severe | 126 | 9,0 |
| Joint/limb pain | None | 936 | 66,7 |
|  | Mild | 187 | 13,3 |
|  | Moderate | 204 | 14,5 |
|  | Severe | 77 | 5,5 |
| Cough | None | 1106 | 78,8 |
|  | Mild | 207 | 14,7 |
|  | Moderate | 80 | 5,7 |
|  | Severe | 11 | 0,8 |
| Rhinitis | None | 1235 | 88,0 |
|  | Mild | 122 | 8,7 |
|  | Moderate | 43 | 3,1 |
|  | Severe | 4 | 0,3 |
| Sore throat | None | 1267 | 90,2 |
|  | Mild | 102 | 7,3 |
|  | Moderate | 31 | 2,2 |
|  | Severe | 4 | 0,3 |
| Abdominal pain | None | 1281 | 91,2 |
|  | Mild | 79 | 5,6 |
|  | Moderate | 36 | 2,6 |
|  | Severe | 8 | 0,6 |
| Diarrhea | None | 1310 | 93,3 |
|  | Mild | 59 | 4,2 |
|  | Moderate | 27 | 1,9 |
|  | Severe | 8 | 0,6 |
| Nausea/vomiting | None | 1315 | 93,7 |
|  | Mild | 60 | 4,3 |
|  | Moderate | 26 | 1,9 |
|  | Severe | 3 | 0,2 |
| Fever | None | 1378 | 98,1 |
|  | Mild | 16 | 1,1 |
|  | Moderate | 9 | 0,6 |
|  | Severe | 1 | 0,1 |
